# Non-linear associations of amyloid-β with resting-state functional networks and their cognitive relevance in a large community-based cohort of cognitively normal older adults

**Published:** 2025-10-14

**Authors:** Junjie Wu, Benjamin B Risk, Taylor A James, Nicholas Seyfried, David W Loring, Felicia C Goldstein, Allan I Levey, James J Lah, Deqiang Qiu

**Affiliations:** 1 Department of Neurology, Emory University, Atlanta, GA, United States; 2 Department of Radiology and Imaging Sciences, Emory University, Atlanta, GA, United States; 3 Department of Biostatistics and Bioinformatics, Emory University, Atlanta, GA, United States; 4 Department of Biochemistry, Emory University, Atlanta, GA, United States; 5 Joint Department of Biomedical Engineering, Emory University and Georgia Institute of Technology, Atlanta, GA, United States

**Keywords:** Alzheimer’s disease, amyloid-β, functional connectivity, default mode network, cognitive aging

## Abstract

**Background:**

Non-linear alterations in brain network connectivity may represent early neural signatures of Alzheimer’s disease (AD) pathology in cognitively normal older adults. Understanding these changes and their cognitive relevance could provide sensitive biomarkers for early detection. Most prior studies recruited participants from memory clinics, often with subjective memory concerns, limiting generalizability.

**Methods:**

We examined 14 large-scale functional brain networks in 968 cognitively normal older adults recruited from the community using resting-state functional MRI, cerebrospinal fluid (CSF) biomarkers (amyloid-β 1–42 [Aβ], total tau, phosphorylated tau 181), and neuropsychological assessments. Functional networks were identified using group independent component analysis.

**Results:**

Inverted U-shaped associations between CSF Aβ and functional connectivity were observed in the precuneus network and ventral default mode network (DMN), but not in the dorsal DMN, indicating network-specific vulnerability to early amyloid pathology. Higher connectivity in Aβ-related networks, including dorsal and ventral DMN, precuneus, and posterior salience networks, was associated with better visual memory, visuospatial, and executive performance. No significant relationships were observed between CSF tau and functional connectivity.

**Conclusions:**

Using a large, community-based cohort, we demonstrate that non-linear alterations in functional connectivity occur in specific networks even during the asymptomatic phase of AD. Moreover, Aβ-related network connectivity is cognitively relevant, highlighting functional brain networks as promising imaging markers for early detection and prognosis of AD.

## 1. Background

Alzheimer’s disease (AD) is a progressive neurodegenerative disorder characterized by the deposition of amyloid-β (Aβ) plaques and neurofibrillary tangles of hyperphosphorylated tau (P-tau) proteins in the brain (1, 2). Clinical symptoms of cognitive impairment can take decades to appear following the onset of AD pathology (1–3). Identifying sensitive biomarkers of this early, asymptomatic stage is of paramount importance, as therapeutic interventions are likely to be most effective when implemented before substantial neurodegeneration occurs (4).

Functional MRI provides a valuable avenue for such early detection, as it can capture subtle synaptic and network-level abnormalities that precede structural atrophy and cognitive decline (1, 5). Changes in intrinsic functional connectivity have emerged as early signatures of AD pathology (6). Aβ deposition has been associated with aberrant connectivity in vulnerable regions such as the hippocampus (7) and default mode network (DMN) (8). Notably, these alterations often follow a biphasic trajectory, with early hyperconnectivity interpreted as compensatory responses, followed by hypoconnectivity as network failure emerges with advancing pathology (9, 10). By contrast, tau pathology appears to exert later and distinct effects on neural activity and connectivity (11–14).

Despite these advances, prior studies in cognitively normal older adults have important limitations. Most have targeted individual networks, such as the DMN (10, 15–20), executive control (15, 16), or salience (10) networks, typically in small samples (*N* < 100). Moreover, many cohorts were recruited from memory clinics, where participants frequently present with subjective memory concerns, raising the possibility of selection bias toward individuals already on a symptomatic trajectory. Consequently, it remains unclear whether biphasic connectivity changes extend beyond the DMN, how they differentially relate to Aβ versus tau pathology, and whether they hold measurable cognitive relevance in large community-based samples of cognitively normal older adults.

The present study addresses these gaps by systematically examining associations between cerebrospinal fluid (CSF) biomarkers of Aβ and tau and intrinsic functional connectivity across 14 large-scale brain networks in 968 cognitively normal individuals recruited from the community. We tested for nonlinear (quadratic) associations to capture potential biphasic trajectories of brain connectivity with AD biomarkers. In addition, we assessed the cognitive significance of these network alterations by correlating functional connectivity with performance on a comprehensive neuropsychological battery spanning memory, language, visuospatial, and executive domains. By integrating large sample size, community-based recruitment, biomarker quantification, and nonlinear modeling, this study provides new insights into the earliest network changes in the AD cascade and their relevance for cognition.

## 2. Methods

### 2.1. Participants

This study included 968 cognitively normal older participants (median age = 63.8 [58.1 – 69.2] years, 641 females [66.2%]) from Emory Healthy Brain Study (21) (Table 1). This Health Insurance Portability and Accountability Act-compliant study was approved by the institutional review board at Emory University School of Medicine. Written informed consent was obtained from all participants prior to study procedures in accordance with the Declaration of Helsinki.

### 2.2. MRI acquisition

MRI data were acquired on a Siemens Magnetom Prisma 3T scanner (Siemens Healthcare, Erlangen, Germany) equipped with a 32-channel head array coil. T_1_-weighted anatomical images were acquired using a magnetization-prepared rapid acquisition with gradient echo (MPRAGE) sequence (TR/TE = 2300/2.96 ms, TI = 900 ms, FA = 9°, voxel size = 1 × 1 × 1 mm^3^, 208 slices). A 10-min resting-state functional MRI was performed using a multiband accelerated gradient-echo echo-planar imaging sequence (TR/TE = 1890/30 ms, FA = 52°, voxel size = 1.5 × 1.5 × 1.5 mm^3^, 81 slices, multiband factor = 3, 320 volumes).

### 2.3. MRI analysis

Preprocessing of functional MRI images was performed using CONN functional connectivity toolbox 22a (https://www.conn-toolbox.org). The functional images were corrected for B0 field inhomogeneity, head motion, and timing of slice acquisition. The resultant images were then normalized to the Montreal Neurological Institute stereotaxic space, spatially smoothed with an 8 mm full-width-at-half-maximum Gaussian kernel, and bandpass-filtered to retain signal components with temporal frequency between 0.01 and 0.1 Hz.

Spatially constrained independent component analysis (ICA) (22) was performed on the functional MRI data to identify functional networks using GIFT toolbox 4.0b (https://trendscenter.org/software/gift). Using the templates from a previous study (23), group ICA was applied to compute ICA components of the following 14 networks: the auditory network, language network, primary visual network, higher visual network, visuospatial network, sensorimotor network, basal ganglia network, precuneus network, anterior salience network, posterior salience network, left executive control network (LECN), right executive control network (RECN), dorsal DMN, and ventral DMN. The networks for each participant were generated using regression-based back-reconstruction (24).

### 2.4. CSF sampling and analysis

CSF was collected in a standardized fashion applying common pre-analytical methods. Lumbar punctures were performed using a 24-g atraumatic Sprotte spinal needle (Pajunk Medical Systems, Norcross, Georgia, USA) with aspiration. After clearing any blood contamination, CSF was transferred into 15-ml polypropylene tubes (Corning, Glendale, Arizona, USA) followed by freezing in 0.5 ml aliquots on dry ice within 1 hour after collection. Aliquots were stored in 0.9 ml FluidX tubes (Azenta, Chemsford, Massachusetts, USA) at −80°C. Following a single freeze-thaw cycle, amyloid-β 1–42 (Aβ), total tau (T-tau), and tau phosphorylated at threonine 181 (P-tau) assays were performed on CSF samples on a Roche Cobas e601 analyzer using the Elecsys immunoassay platform (25). All assays were performed in a single laboratory in the Emory Goizueta Alzheimer’s Clinical Research Unit. P-tau/Aβ ratio was calculated as an indicator of Aβ and tau burden.

### 2.5. Neuropsychological assessments

A neuropsychological test battery was administered, including the Rey Complex Figure Test (RCFT) (26) for visual memory and visuospatial functioning, the Judgment of Line Orientation (JoLO) (27) for visuospatial ability, the Rey Auditory Verbal Learning Test (RAVLT) (26) for verbal learning and memory, the Letter Fluency (FL) (28) for language and executive functioning, the Animal Fluency (28) for language and semantic memory, the Trail Making Test Part A (TMTA) (29) for processing speed, and the Trail Making Test Part B (TMTB) (29) for executive functioning.

### 2.6. Statistical analysis

Associations of functional connectivity with CSF biomarker measurements were estimated using multiple regression including quadratic terms for CSF biomarkers, with age and sex as covariates. Correlations between functional connectivity and neuropsychological performance were evaluated using multiple regression with age and sex as covariates. The statistical analyses used a two-tailed level of 0.05 for defining statistical significance, and the Benjamini-Hochberg false discovery rate (FDR) procedure was applied to correct for multiple testing.

## 3. Results

**Fig. 1.**
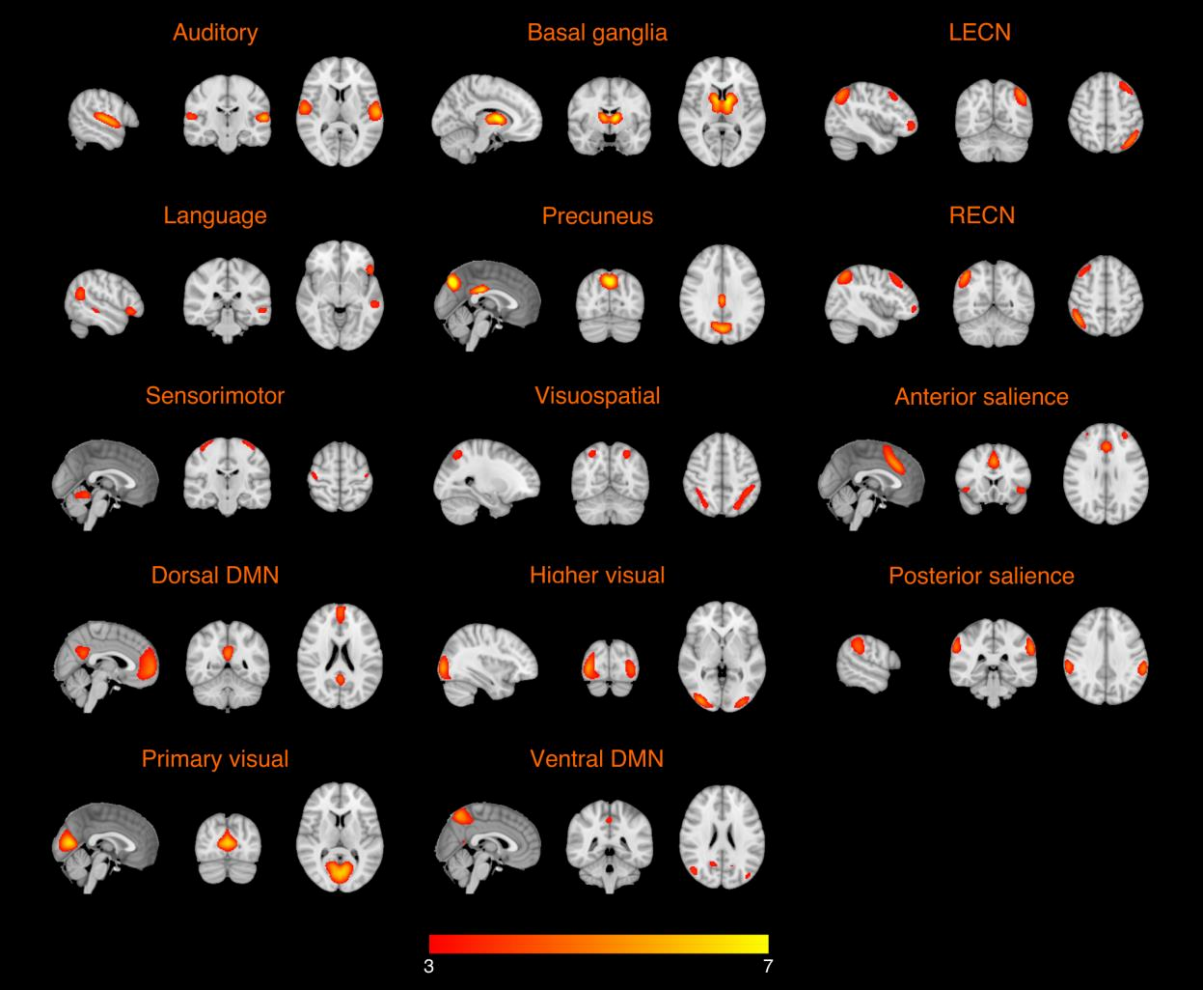
Averaged connectivity maps across all healthy older adults for the auditory network, basal ganglia network, left executive control network (LECN), language network, precuneus network, right executive control network (RECN), sensorimotor network, visuospatial network, anterior salience network, dorsal default mode network (DMN), higher visual network, posterior salience network, primary visual network, and ventral DMN.

Fig. 1 shows mean connectivity maps across all participants for the 14 functional networks.

**Fig. 2.**
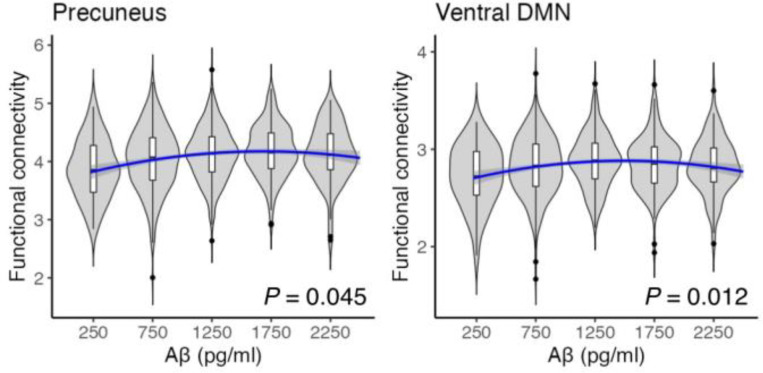
Violin plots illustrating non-linear associations between cerebrospinal fluid (CSF) amyloid-β 1–42 (Aβ) levels and functional connectivity in the precuneus network and ventral default mode network (DMN). Distributions of functional connectivity are displayed across the range of Aβ values, with quadratic associations indicated by blue curves and shaded areas representing the 95% confidence intervals. Associations were evaluated using multiple regression controlling for age and gender, with *P* values corrected using the false discovery rate.

Fig. 2 presents correlations between CSF biomarkers and functional networks. In the precuneus network, both the linear (*β* = 0.126, 95% CI: 0.058 – 0.193, *P* = 0.004, FDR corrected) and quadratic (*β* = −0.071, 95% CI: −0.122 to −0.020, *P* = 0.045, FDR corrected) terms for Aβ were significant, indicating a non-linear (inverted U-shaped) relationship between Aβ levels and functional connectivity. In the ventral DMN, the quadratic term for Aβ was also significant (*β* = −0.089, 95% CI: −0.141 to −0.037, *P* = 0.012, FDR corrected), whereas the linear term was not significant (*β* = 0.079, 95% CI: 0.010 – 0.148, *P* = 0.119, FDR corrected). These findings suggest that non-linear associations between CSF Aβ and functional connectivity are evident across multiple large-scale networks. Complementary scatter plots of the raw data with fitted curves are provided in Supplementary Fig. S1.

**Fig. 3.**
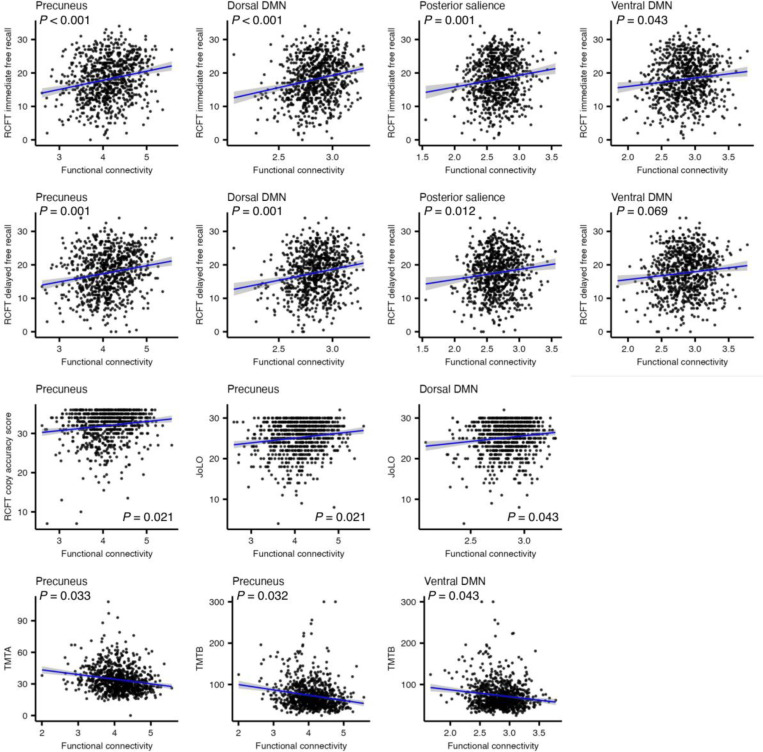
Correlations between functional connectivity and neuropsychological performance. Functional connectivity in the precuneus, dorsal default mode network (DMN), posterior salience network, and ventral DMN was positively associated with Rey Complex Figure Test (RCFT) immediate free recall. RCFT delayed free recall was positively associated with connectivity in the precuneus, dorsal DMN, and posterior salience networks, with a trend toward significance for the ventral DMN. RCFT copy accuracy was positively associated with precuneus connectivity. Visuospatial ability, measured by the Judgment of Line Orientation (JoLO), was positively associated with connectivity in the precuneus and dorsal DMN. Processing speed and executive functioning, measured by the Trail Making Test Part A (TMTA) and Part B (TMTB), were faster with higher connectivity in the precuneus and ventral DMN. Linear associations are indicated with blue regression lines, and shaded areas represent the 95% confidence intervals. Associations were evaluated using multiple regression controlling for age and gender, and *P* values were corrected using the false discovery rate.

Functional connectivity was correlated with performance across multiple cognitive domains (Fig. 3). In visual memory and visuospatial functioning, higher connectivity in the precuneus (β = 0.168, 95% CI: 0.099 – 0.236, *P* < 0.001, FDR corrected), dorsal DMN (β = 0.168, 95% CI: 0.099 – 0.236, *P* < 0.001, FDR corrected), posterior salience (β = 0.141, 95% CI: 0.074 – 0.208, *P* = 0.001, FDR corrected), and ventral DMN (β = 0.100, 95% CI: 0.033 – 0.168, *P* = 0.043, FDR corrected) networks was associated with better RCFT immediate recall. RCFT delayed recall showed similar associations in the precuneus (β = 0.147, 95% CI: 0.078 – 0.216, *P* = 0.001, FDR corrected), dorsal DMN (β = 0.147, 95% CI: 0.078 – 0.216, *P* = 0.001, FDR corrected), and posterior salience (β = 0.120, 95% CI: 0.052 – 0.188, *P* = 0.012, FDR corrected) networks, with a trend toward significance for ventral DMN (β = 0.093, 95% CI: 0.025 – 0.161, *P* = 0.069, FDR corrected). Precuneus connectivity also correlated positively with RCFT copy accuracy (β = 0.116, 95% CI: 0.046 – 0.186, *P* = 0.021, FDR corrected). For visuospatial ability, higher connectivity in the precuneus (β = 0.113, 95% CI: 0.046 – 0.181, *P* = 0.021, FDR corrected) and dorsal DMN (β = 0.099, 95% CI: 0.031 – 0.166, *P* = 0.043, FDR corrected) was associated with better JoLO performance. For processing speed and executive functioning, faster completion times on TMTA and TMTB were linked to higher connectivity in the precuneus (TMTA: β = −0.100, 95% CI: −0.165 to −0.036, *P* = 0.033, FDR corrected; TMTB: β = −0.100, 95% CI: −0.164 to −0.037, *P* = 0.032, FDR corrected) and ventral DMN (TMTB: β = −0.091, 95% CI: −0.153 to −0.028, *P* = 0.043, FDR corrected). An additional association between higher visual network connectivity and poorer RCFT recognition performance was observed (Supplementary Fig. S2).

## 4. Discussion

In this large, community-based cohort of cognitively normal older adults, we provide novel insights into the earliest network changes in the AD cascade and their cognitive relevance. Specifically, inverted U-shaped associations between CSF Aβ and functional connectivity were observed in the precuneus network and ventral DMN, but not in the dorsal DMN. Higher connectivity in Aβ-related networks, including the dorsal and ventral DMN, precuneus, and posterior salience networks, was associated with better visual memory, visuospatial, and executive performance.

Converging evidence indicates that Aβ preferentially accumulates in the DMN and salience network (10, 30). Although Aβ pathology has been previously reported to be associated with non-linear alterations in functional connectivity in DMN (9, 10), our results further demonstrate that these relationships are evident specifically in the precuneus network and ventral DMN, but not in the dorsal DMN. This network-specific vulnerability suggests that early Aβ deposition preferentially affects the precuneus and medial temporal subsystem of the DMN, including the hippocampus. In contrast, the dorsal DMN may be less sensitive to early Aβ effects due to its relatively lower metabolic demand or later involvement in the disease cascade (8, 31). These findings underscore the importance of examining DMN subsystems separately rather than treating the network as a single entity.

We did not observe significant associations between CSF tau (P-tau 181 or T-tau) and functional connectivity across any network. This is consistent with prior evidence indicating that tau pathology has later effects on network connectivity (11). Notably, CSF P-tau 181 showed a stronger correlation with amyloid PET than with tau PET (32), which may obscure independent effects of tau on functional connectivity during the asymptomatic phase. Future studies incorporating tau PET imaging will be important to clarify the relationship between functional networks and tau in cognitively normal older adults.

AD-related cognitive impairment is reported in diverse domains, including memory, attention, executive function, language and visuospatial ability (30). In the present study, higher connectivity within Aβ-related brain networks (10, 30), i.e., dorsal and ventral DMN, precuneus, and posterior salience networks, was associated with better neuropsychological performance, particularly in visual memory, visuospatial, and executive domains. These findings suggest that even subtle network disruptions during the asymptomatic phase of AD may have measurable cognitive relevance.

Several limitations should be noted. First, our sample included a disproportionately higher number of female participants, reflecting women’s greater willingness to participate in research. This imbalance may limit representativeness, and future studies should strive for a more balanced sex distribution to improve generalizability. Second, while this cross-sectional study benefits from a large sample size, longitudinal evaluation of changes in brain network connectivity is necessary to better understand the temporal dynamics of network alterations. Follow-up data are currently being collected as part of the ongoing Emory Healthy Brain Study.

## 5. Conclusions

In conclusion, this study provides novel evidence that early Aβ pathology is associated with non-linear alterations in functional connectivity within the precuneus network and ventral DMN, but not the dorsal DMN. Aβ-related brain networks, including the dorsal and ventral DMN, precuneus, and posterior salience networks, support visual memory, visuospatial, and executive performance. Functional brain networks may serve as sensitive imaging markers for the early detection and prognosis of AD.

## Figures and Tables

**Table 1.** Demographics, CSF, and neuropsychological assessments

	Median [Q1 – Q3] / n (%)
N	968
Age (years)	63.8 [58.1 – 69.2]
Sex (female)	641 (66.2%)
Aβ (pg/ml)	1197.0 [847.1 – 1555.2]
T-tau (pg/ml)	163.1 [127.9 – 212.5]
P-tau (pg/ml)	14.2 [10.9 – 18.5]
P-tau/Aβ ratio	0.011 [0.009 – 0.015]
RCFT immediate free recall	18.0 [13.5 – 23.0]
RCFT delayed free recall	18.0 [13.0 – 22.5]
Recognition of RCFT elements	21.0 [19.0 – 22.0]
RCFT copy accuracy score	33.0 [31.0 – 35.0]
JoLO	26.0 [23.0 – 28.0]
RAVLT delayed recall	10.0 [6.0 – 12.0]
Letter Fluency (FL)	29.0 [24.0 – 34.0]
Animal Fluency	21.0 [18.0 – 25.0]
TMTA	32.0 [26.0 – 40.0]
TMTB	65.0 [52.0 – 84.0]

RCFT = Rey Complex Figure Test; JoLO = Judgment of Line Orientation; RAVLT = Rey Auditory Verbal Learning Test; TMTA = Trail Making Test Part A; TMTB = Trail Making Test Part B.

## Data Availability

The data that support the findings of this study are available upon reasonable request from qualified investigators, adhering to ethical guidelines and signing a data use agreement with the authors’ institution.

## Abbreviations

AD: Alzheimer’s disease
Aβ: amyloid-β
P-tau: hyperphosphorylated tau
DMN: default mode network
CSF: cerebrospinal fluid
ICA: independent component analysis
LECN: left executive control network
RECN: right executive control network
T-tau: total tau
RCFT: Rey Complex Figure Test
JoLO: Judgment of Line Orientation
RAVLT: Rey Auditory Verbal Learning Test
FL: Letter Fluency
TMTA: Trail Making Test Part A
TMTB: Trail Making Test Part B
FDR: false discovery rate
